# The effects of metabolites from three vaginal bacteria on the Syndecan-1 of cervical epithelial cells

**DOI:** 10.1016/j.heliyon.2024.e33426

**Published:** 2024-06-22

**Authors:** Yan Xia, Ying Feng, Lan Jiang, Youqiang Heng, Xiaoqin Li, Cailing Ma

**Affiliations:** a Department of Assisted Reproduction, Urumqi Maternal and Child Health Hospital, Urumqi, 830000, China; b Department of Gynecology, The First Affiliated Hospital of Xinjiang Medical University, State Key Laboratory of Pathogenesis, Prevention and Treatment of High Incidence Diseases in Central Asia, Urumqi, 830000, China; c Department of Laboratory Medicine, Urumqi Maternal and Child Health Hospital, Urumqi, 830000, China; d Xinjiang Key Laboratory of Medical Animal Model Research, Urumqi, 830000, China

**Keywords:** Human papillomavirus, Syndecan 1, Cervical epithelial cells

## Abstract

This study aims to explore the impact of metabolites from three vaginal bacteria on the expression of Syndecan 1 (SDC-1). Human cervical epithelial cells (HcerEpic) were separately incubated with the cell-free supernatants of *Lactobacillus crispatus* (LCS group), *Gardnerella vaginalis* (GVS group), and *Atopobium vaginalis* (AVS group). LCS showed a proliferative effect on HcerEpic, with the most significant effect observed at a concentration of 30 % (P < 0.001). GVS and AVS exhibited some cytotoxicity, with significant growth inhibitory effects observed at concentrations of 30 % and 40 % (P < 0.01). Therefore, subsequent experiments were conducted using 30 % LCS, 40 % GVS, and 40 % AVS. In terms of cellular morphology, compared to the Control group, the LCS group showed more frequent fusion of cell sheets, with no obvious changes in the morphology of individual cells. In the GVS and AVS groups, some individual cells became round and smaller, with reduced protrusions and even a small amount of floating cells. The metabolic products of the three vaginal bacteria significantly upregulated the expression of IL-1β, IL-6, and TNF-α in HcerEpic (P < 0.05). In the GVS and AVS groups, the level of SDC-1 on the surface of HcerEpic was significantly decreased (P < 0.01), while the concentration of SDC-1 in the cell culture supernatant was significantly increased (P < 0.0001). Additionally, the level of *SDC-*1 mRNA was significantly downregulated (P < 0.01). In the LCS group, no significant changes were observed in SDC-1 protein and mRNA expression (P > 0.05). LCS promotes HcerEpic proliferation, without significant impact on SDC-1 expression and shedding. This provides molecular evidence for LCS as a protective factor against human papillomavirus infection in the cervix. Metabolites of GV and AV inhibit HcerEpic proliferation, induce cytokine secretion, suppress SDC-1 transcription and expression, and promote SDC-1 shedding.

## 1 Introduction

The persistence of human papillomavirus (HPV) infection is a crucial factor in the development of cervical cancer [1]. Controlling HPV infection can help decrease the incidence of cervical cancer. Present research evidence indicates that the vaginal microbiota is a novel factor in the natural progression of HPV infection [2]. The dysbiosis of microorganisms in the female reproductive system increases susceptibility to viruses and bacteria [3,4]. Oral intake of *Lactobacillus crispatus* M247 can improve the vaginal microbiota and enhance HPV clearance [5]. As the severity of cervical abnormalities increases, there is a decrease in *Lactobacillus* abundance (relative abundance less than 50 %) and an increase in anaerobic bacteria abundance [6]. However, the specific role of vaginal bacteria in HPV infection remains unclear.

The process of HPV infection in cells involves adhesion, endocytosis, and nuclear transport. The extracellular matrix receptor, syndecan 1 (SDC-1), a major protein that serves as the primary receptor for HPV adhesion, has been shown to play a significant role in HPV infection [7,8]. SDC-1 is structured with an extracellular domain, a highly conserved transmembrane region, and an intracellular domain. The extracellular domain is linked to both a chondroitin sulfate chain and at least one heparan sulfate chain. Lipopolysaccharide and matrix metalloproteinases in the extracellular matrix can induce the complete shedding of the extracellular domain [9,10]. This shedding process results in the formation of soluble SDC-1, while the uncleaved form is known as membrane-bound SDC-1. Culp et al. found that HPV capsids could adsorb extracellular matrix components secreted by keratinocytes, and HPV-11 could adsorb and reinfect keratinocytes through extracellular matrix receptors [11]. Moreover, Surviladze et al. [12] demonstrated that soluble SDC-1 competed with membrane-bound SDC-1 in binding to HPV, thereby forming SDC-1-HPV complexes in the extracellular matrix. These complexes enhance HPV's ability to infect keratinocytes and can even infect cells that do not express SDC-1 [13]. Inhibiting the shedding of SDC-1 has been shown to decrease HPV infection in human keratinocytes [13]. Recently, Cheudjeu et al. [14] also suggested that HPV infection was accompanied by down-regulation of glycocalyces, decreased expression of glycoproteins on the cell surface, and increased endocytosis of HPV-16 and HPV-31 viruses, along with SDC shedding, especially when heparinase was overexpressed.

Our previous study [15] demonstrated that the vaginal microbiota of asymptomatic women of childbearing age in Xinjiang, China, who were infected with HPV, exhibited an overabundance of *Gardnerella vaginalis* (GV) (the relative abundance increased from 5.3 % to 11.1 %) and a decrease in *Lactobacillus* species (the relative abundance decreased from 89.87 % to 72.14 %). The three most abundant genera were *Lactobacillus*, *Gardnerella*, and *Atopobium*. Similarly, Fang et al. [16] reported that at the genus level, *Lactobacillus* accounted for 84.90 % of the Control group, followed by *Gardnerella* (1.76 %) and *Atopobium* (0.21 %). However, in the high-risk HPV group, the relative abundance of *Lactobacillus* decreased to 54.98 %, with *Gardnerella* at 11.59 % and *Atopobium* at 3.43 %. In this study, we selected metabolites derived from *Lactobacillus crispatus* (LC), GV, and *Atopobium vaginalis* (AV) based on the analysis of clinical samples. These metabolites were used to intervene with HcerEpic cells, aiming to evaluate their influence on SDC-1 expression. Our findings may provide experimental evidence for the prevention of HPV infection.

## 2 Materials and methods

### 2.1 Bacterial strains and culture conditions

The bacterial strains LC (ATCC 33820), AV (ATCC-BAA-55), and GV (ATCC 14018) were purchased from the American Type Culture Collection (ATCC, Manzas, Virginia, USA). They were all inoculated on Columbia blood agar plates (LS0109, Guangzhou Dijing Microbial Technology Co., Ltd., China). LC and GV were incubated at 35 °C under humid conditions with 5 % CO2 for 48 h, while AV was incubated at 35 °C in a completely anaerobic environment for 72 h.

### 2.2 Extraction of bacterial metabolites

After the formation of colonies on the agar plate, the bacterial suspension was prepared with the turbidity adjusted to 1.0 using a McFarland turbidity meter (DensiCHEK plus, Biomerieux, France). Then, 10 mL of the suspension was collected and centrifuged at 3500 rpm for 10 min. The supernatant was discarded. Subsequently, the bacterial cells were washed with 5 mL of PBS, followed by the addition of 5 mL of DMEM high-glucose medium (Hyclone, USA) containing 10 % fetal bovine serum (F7524, Sigma, USA). The mixture was thoroughly mixed by pipetting and incubated at 35 °C. After 4 h, the mixture was centrifuged at 3500 rpm for 10 min. The supernatant was subjected to repeated centrifugation and the filtrate, which contained bacterial metabolites, was filtered through a 0.22 μm disposable filter. Finally, the filtrate was stored at −80 °C for further use.

### 2.3 Cell lines and culture

Human cervical epithelial cells (HcerEpic; ScienCell, San Diego, CA, USA) were cultured in DMEM medium containing 10 % fetal bovine serum (FBS) and 1 % penicillin-streptomycin (10000 U/ml, Gibco, Grand Island, NY, USA) under humidified conditions at 37 °C and 5 % CO2. According to different treatments, the cells were divided into four groups, including the control group, the LC supernatant (LCS) group, the GV supernatant (GVS) group, and the AV supernatant (AVS) group. After the addition of bacterial culture supernatant, the cells were cultured for 24 h.

### 2.4 Cell viability detection and morphological change observation

The Cell Counting Kit (CCK-8, BA00208, Boster Biological Technology Co., Ltd., Wuhan, China) was used to evaluate the effects of different concentrations of bacterial metabolites on the viability of HcerEpic cells. As previously reported [17], the concentrations of three types of bacterial metabolites (filtrate volume/total culture solution volume ratio) were set as 20 %, 30 %, and 40 %. After 24 h of culture, the CCK-8 reagent was added and incubated at 37 °C for 1 h, followed by measuring the optical density at 450 nm (OD450) using a microplate reader. Cell viability was calculated by (OD450 experimental group - OD450 blank group)/(OD450 control group - OD450 blank group) × 100 %. Simultaneously, morphological changes in the four groups of cells were observed under an optical microscope (DMI8, Leica, Germany).

### 2.5 ELISA

To assess the impact of various bacterial metabolites on the secretion of inflammatory cytokines by HcerEpic, we measured the levels of IL-1β, IL-6, and TNF-α in the cell culture supernatant after 24 h of pretreatment, following the instructions provided with the ELISA kits (MULTISCIENCES(LIANKE) BIOTECH, CO., LTD, Hangzhou, China). We also measured the SDC-1 level in the supernatant, using the SDC-1 assay kit (Wuhan Huamei Biotechnology Co., Ltd., China). Briefly, the microplate was incubated with the corresponding samples for 2 h at room temperature. Subsequently, the incubation with the antibodies was performed at room temperature for 45 min. Finally, TMB chromogenic solution was used for color development. The absorbance was detected by a microplate reader.

### 2.6 Flow cytometry

Flow cytometry detected the expression of SDC-1 on the cell surface [18,19]. Briefly, cells were digested with 0.25 % trypsin, collected, and centrifuged at 1000 rpm for 5 min. The cell pellet was resuspended in 1 % sodium azide PBS solution containing 10 % FBS. Then, 100 μL of the cell suspension was incubated with 100 μL of SDC-1 antibody (1:100; ab181789, Abcam, Cambridge, UK) in a dark environment at 4 °C for 30 min. After washing three times with cold PBS, the cells were incubated in 200 μL of goat anti-mouse IgG H&L (1:2000; ab150113, Abcam) in the dark at 4 °C for another 30 min. Following incubation, the cells were resuspended in 200 μL of 1 % sodium azide PBS solution containing 3 % FBS. The cell suspension was then filtered through a 200-mesh sieve and evaluated with a flow cytometer (LSRFortessa, BD, USA) under the Alexa Fluor 488 channel within 1 h. The unstained samples with an average fluorescence intensity of around 89 were also observed. For each group, 6 samples were run, and approximately 10,000 cells were observed in each sample. Dead cells were not removed; however, a gate was set in the scatter plot to count only the target cells, and the mean fluorescence intensity reported was specifically for positive cells.

### 2.7 Real-time quantitative PCR (RT-qPCR)

Total RNA was extracted from HcerEpic cells using the Trizol reagent (Ambion, Austin, TX, USA). The extracted RNA was subsequently reverse-transcribed into cDNA using the 5X All-in-one MasterMix kit (G592, ABM, Vancouver, Canada), which also facilitated genomic DNA removal. The cDNA samples were then subjected to RT-qPCR analysis using the BlasTaqTM 2 × qPCR MasterMix kit (G891, ABM) as per the manufacturer's instructions. The primer sequences were: SDC-1 Forward CTGGACAGGAAAGAGGTGCT and SDC-1 Reverse TGTTTGGTGGGCTTCTGGTA; and, GAPDH Forward TGTTGCCATCAATGACCCCTT and GAPDH Reverse CTCCACGACGTACTCAGCG. GAPDH served as an internal reference gene. The reaction conditions for RT-qPCR were as follows: initial denaturation at 95 °C for 3 min, followed by 40 cycles of denaturation at 95 °C for 15 s, and annealing/extension at 60 °C for 60 s. The relative gene expression was calculated with the 2^−ΔΔCt^ method.

### 2.8 Statistical analysis

SPSS 22.0 and GraphPad Prism 8.0 software were used for data analysis and graph plotting. The data are presented as mean ± standard deviation. Independent samples *t*-test was used for comparisons between two groups, while one-way analysis of variance (ANOVA) was used for multiple comparisons. A p-value less than 0.05 was considered statistically significant.

## 3 Results

### 3.1 Different viabilities of HcerEpic treated with three vaginal bacterial metabolites

**Fig. 1:**
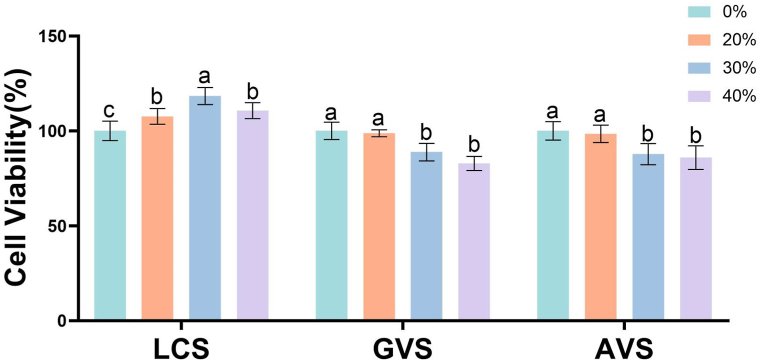
**Effects of different concentrations of metabolites from three vaginal bacteria on the viability of HcerEpic cells.** HcerEpic cells were treated with LCS, GVS, and AVS at concentrations of 0 %, 20 %, 30 %, and 40 % for 24 h, and the cell viability of each group was measured using the CCK-8 assay. The experiment was repeated three times, and the results are presented as mean ± standard deviation. In the bar chart, differently labeled letters in different concentrations of the three vaginal bacteria indicated significant differences (lowercase letters P < 0.05).

HcerEpic was stimulated with metabolites of different concentrations (v/v), including 20 %, 30 %, and 40 %. After incubation for 24 h, the cell viability of HcerEpic was assessed using the CCK-8 assay. The results showed that LCS had a proliferative effect on HcerEpic cells, with the most significant effect observed at a concentration of 30 % (P < 0.001) (Fig. 1). GVS and AVS exhibited certain cytotoxicity towards HcerEpic cells, showing concentration-dependent inhibition, with the strongest effect observed at a concentration of 40 % (P < 0.01). However, the cell viability remained above 80 %. Therefore, subsequent experiments were conducted using 30 % LCS, 40 % GVS, and 40 % AVS as stimulation agents.

### 3.2 Morphological changes of HcerEpic treated with three vaginal bacterial metabolites

**Fig. 2:**
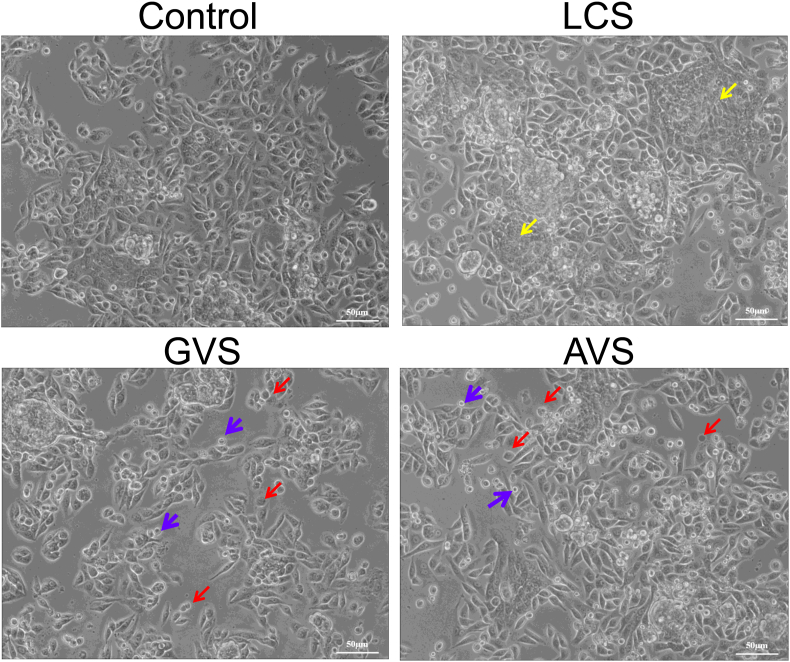
**Effects of metabolites from three vaginal bacteria on the morphology of HcerEpic cells.** Yellow arrows indicate cell fusion, red arrows represent round cells with shortened pseudopodia, and purple arrows highlight reduced cell volume and pyknosis.

HcerEpic cells were stimulated with 30 % LCS, 40 % GVS, and 40 % AVS for 24 h, and changes in cellular morphology were observed under an optical microscope. As shown in Fig. 2, the individual HcerEpic cells in the Control group exhibited a spindle-shaped morphology, with elongated and short cytoplasmic protrusions, along with polygonal-shaped fused cells. In the LCS group, the HcerEpic cells displayed the same morphology as the Control group, but with a higher occurrence of patchy fusions. Conversely, in the GVS and AVS groups, a subset of HcerEpic cells demonstrated noticeably reduced cell body sizes, circular shapes, and the absence of cytoplasmic protrusions. Additionally, a few contracted cells were observed floating. Thus, the metabolites from three vaginal bacteria can affect the morphology of HcerEpic.

### 3.3 The metabolites from three vaginal bacteria elevate the secretion of inflammatory factors in HcerEpic cells

**Fig. 3:**
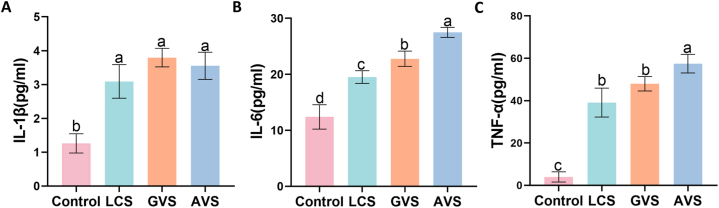
**Effects of metabolites from three vaginal bacteria on the secretion of inflammatory factors in HcerEpic cells.** The inflammatory factors in the supernatant of HcerEpic cells of each group were measured with ELISA. A: Concentration of IL-1β; B: Concentration of IL-6; C: Concentration of TNF-α. The experiment was repeated three times, and the results are presented as mean ± standard deviation. Different letters represent significant differences (lowercase letters P < 0.05).

The inflammatory factors IL-1β, IL-6, and TNF-α are all secretory proteins. The concentrations of IL-1β, IL-6, and TNF-α in the supernatant of four groups of cell cultures were detected using ELISA. Fig. 3 shows that the metabolites from the three vaginal bacteria significantly stimulate HcerEpic cells. Compared with the control group, the levels of IL-1β (Fig. 3A), IL-6 (Fig. 3B), and TNF-α (Fig. 3C) were significantly increased in the LCS group, GVS group, and AVS group (P < 0.001). Although the IL-1β level was slightly higher in the GVS and AVS groups compared to the LCS group, there was no significant difference among the three groups (P > 0.05). The levels of IL-6 and TNF-α in the GVS and AVS groups were significantly higher than the LCS group (P < 0.05). The levels of IL-6 and TNF-α were the highest in the AVS group, significantly higher than the LCS group and GVS group (P < 0.05). Therefore, GVS and AVS had a stronger effect on the release of inflammatory factors from HcerEpic than LCS.

### 3.4 The vaginal bacterial metabolites facilitate SDC-1 shedding from HcerEpic

**Fig. 4:**
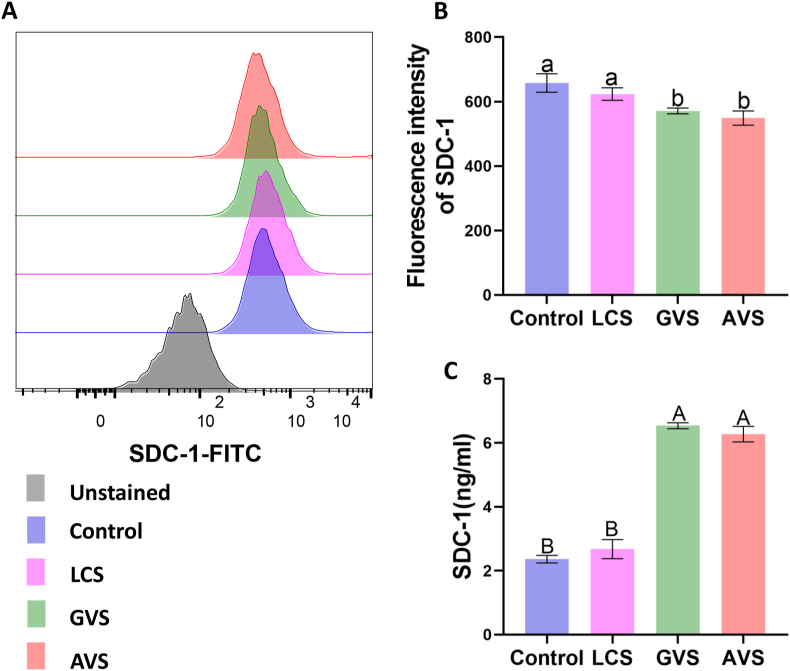
**Effects of metabolites from three vaginal bacteria on the expression of SDC-1 in HcerEpic cells.** A: Flow cytometry detected the expression of SDC-1 on the cell surface. Representative flow cytometry results were shown. B: Column chart representing the quantitative results of flow cytometry. C: Concentration of SDC-1 in the culture supernatant of the four groups of cells as detected by ELISA. The experiment was repeated three times, and the results are presented as mean ± standard deviation. Different letters represent significant differences (lowercase letters P < 0.05, capital letters P < 0.01).

Flow cytometry was used to quantitatively detect the expression of SDC-1 on the cell membrane, while ELISA was used to determine the shedding of SDC-1 in the cell culture supernatant. The results demonstrated a slight decrease in the fluorescence intensity of SDC-1 membrane expression in the LCS group (Fig. 4A and B), and a slight increase in the level of shed SDC-1 (Fig. 4C), compared to the Control group. However, these differences were not statistically significant (P > 0.05). In response to GVS and AVS stimulation, the membrane expression of SDC-1 was significantly downregulated (P < 0.01) compared to both the Control and LCS groups (Fig. 4A and B), whereas the level of SDC-1 shedding exhibited a significant increase (P < 0.0001) (Fig. 4C). Therefore, GVS and AVS were more effective in facilitating SDC-1 shedding from HcerEpic than LCS.

### 3.5 The metabolites from three vaginal bacteria inhibit *SDC-*1 mRNA expression in HcerEpic

**Fig. 5:**
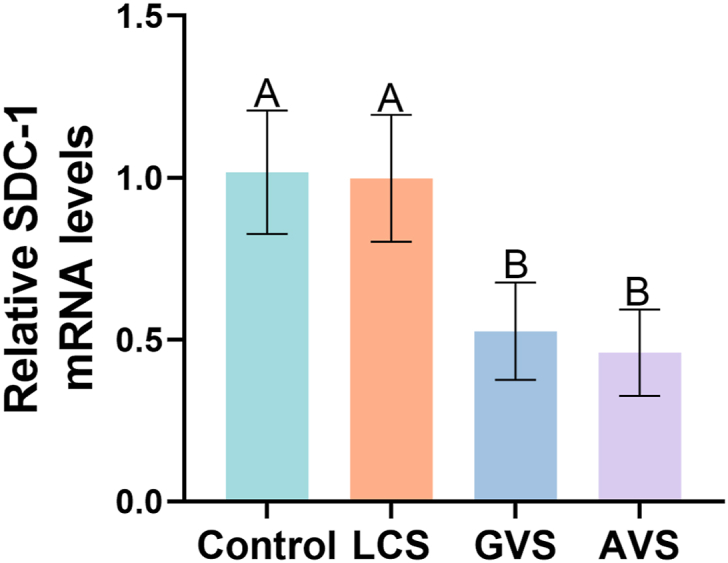
**Effects of metabolites from three vaginal bacteria on the mRNA expression of *SDC-1* in HcerEpic cells.** Relative expression levels of *SDC-*1 mRNA in the four groups were measured using RT-qPCR. The experiment was repeated three times, and the results are presented as mean ± standard deviation. Different letters represent significant differences (capital letters P < 0.01).

Based on the effect of metabolites from three vaginal bacteria on SDC-1 expression in HcerEpic, we further explored the relative expression level of *SDC-*1 mRNA in HcerEpic by using RT-qPCR. The results showed that compared to the Control group, there was no significant change in the expression level of *SDC-*1 mRNA in the LCS group (P < 0.05) (Fig. 5). However, both GVS and AVS significantly downregulated the *SDC-*1 mRNA (P < 0.01).

## 4 Discussion

This study demonstrated that metabolites from three vaginal bacteria had different effects on the vitality and morphology of cervical epithelial cells. Takada et al. [20] intervened with vaginal epithelial cells MS74 using LC, LCS, and heat-inactivated LC, and found that, except heat-inactivated LC, LC and LCS could promote the reepithelialization of MS74, facilitate the rapid healing of damaged vaginal epithelial cells, and maintain the integrity of the vaginal epithelial barrier. Consistently, we observed that there were no evident morphological changes in the HcerEpic of the LCS group, indicating that LC, which is associated with vaginal health, could promote the proliferation of normal epithelial cells. Anton et al. [21] observed that morphological changes in cells such as envelope folds and cell death were more pronounced in the presence of GV than in LC. The impact of GV on cell vitality showed dose dependency. There are relatively few research reports on AV. Doerflinger et al. [22] used AV and LC to infect vaginal epithelial cells at a multiple of infection of 10 particles/cell and observed that compared to LC, AV tended to inhibit cell growth after cell adhesion. Similarly, we found that the cell viability of HcerEpic cells decreased under GVS and AVS stimulation, and the degree of decrease was concentration-dependent. Xiang et al. [23] found that GVS activated the NLRP3 inflammasome in macrophages and human monocytic leukemia cells, inducing cell pyroptosis. In this study, we also observed the toxic effect of GVS and AVS on cervical epithelial cells.

The results of this study showed that under normal conditions, HcerEpic secreted a small amount of IL-1β, IL-6, and TNF-α. However, under the stimulation of vaginal bacteria metabolites, the levels of IL-1β, IL-6, and TNF-α secreted by HcerEpic all significantly increased. Among them, there was no significant difference in the levels of IL-1β between the three groups, but the level of IL-1β secreted by HcerEpic in the GVS group was the highest. This is consistent with a clinical study [24] showing that the levels of IL-1β, TNF-α, and IL-6 in vaginal lavage fluid of women with BV were significantly higher than those of healthy women, and the level of IL-1β was correlated with the number of GV under high-power microscopy. On the other hand, we found that the levels of IL-6 and TNF-α increased significantly in both the GVS group and the AVS group, with the highest level in the AVS group. This is similar to previous research results [22,25]. Compared to *Prévotella bivia* infection of three-dimensional human vaginal epithelial cells or GV infection of cervical epithelial cells, AV can induce a more intense pro-inflammatory immune response, causing vaginal epithelial cells to secrete a large amount of IL-6 and TNF-α. In contrast, whether it is live LC infection or LCS stimulation of cervical epithelial cells, the stimulation is milder compared to GV or GVS, and the levels of IL-6 and TNF-α are significantly lower [21]. This is consistent with the phenomenon that LC is often a common dominant bacterium in the vaginal microbiota of healthy women.

The results of this study showed that the metabolites of three bacterial strains all reduced the expression level of membrane-type SDC-1 in HcerEpic cells. The LCS group showed a slight decrease compared to the control group, while the GVS and AVS groups showed a significant decrease. Furthermore, we observed that the soluble SDC-1 levels in the GVS and AVS groups were more than twice as high as those in the control group. Additionally, the expression of *SDC-*1 mRNA was inhibited. We believe that soluble SDC-1 may originate from the shedding of membrane-type SDC-1, rather than excessive synthesis. This may be related to the strong inflammatory response induced by the metabolites, leading to the shedding of SDC-1. This result is similar to that by Zhang et al. [26] in mucosal sections of patients with ulcerative colitis, as well as the findings of Andrian et al. [9] in gingival epithelial cells, which may be related to the presence of lipopolysaccharide in GVS and AVS, promoting matrix metalloproteinase expression and enhancing the shedding of SDC-1.

The HcerEpic cell line utilized in this research was acquired from human uterine tissue by the Sciencell Research Laboratory in the United States. To induce immortalization, it was transfected with the Simian virus 40, resulting in the production of the Simian virus 40 large T antigen. The interaction between this antigen and the intracellular tumor suppressor gene P53 allows for the sustained survival and proliferation of these cells [27]. HcerEpic cells maintain the morphology and characteristics of cervical epithelial cells, while not expressing the E6/E7 protein. Thus, they serve as a valuable tool for studying benign cervical lesions. Unlike cervical epithelial cells such as HeLa and Caski, which harbor HPV genes, our study used HPV-negative human cervical epithelial cells, which could closely reflect the pathogenesis of normal cells within the human body. No significant alterations were observed in SDC-1 within the LCS group, further affirming that LC, an advantageous bacterium in a healthy vagina, does not exhibit any detrimental effects on cervical epithelial cells. The slight upregulation of inflammatory factors secreted by LC may be linked to the maintenance of a normal immune response within the vaginal environment. Although the AV strain was isolated and cultured from the vaginal flora of healthy women, its impact on cervical epithelial cells mirrors that of the GV strain, which was obtained from women with bacterial vaginosis. Consequently, AV warrants increased attention in both clinical practice and future research.

Bacteria can produce various substances through catabolism and anabolism [28]. During the growth within the human body, bacteria produce multiple metabolites that may interact with cells in competitive or cooperative ways [29]. As a result, definitive metabolite screening was not conducted in this study. An example of a common metabolite found in Gram-negative bacteria is lipopolysaccharide, which typically induces the increased secretion of pro-inflammatory factors in epithelial cells and facilitates SDC-1 shedding [30]. Our experimental findings in the GVS/AVS group were consistent with those of lipopolysaccharide, suggesting that lipopolysaccharide may be a predominant component in the metabolites of GV and AV. Lactic acid, a metabolite produced by LC, is also present in the metabolites of AV [31]. However, the overall metabolite profiles of these two bacteria exert distinct effects on HcerEpic. Notably, Motevaseli et al. indicated that lactic acid was not significantly implicated in these effects [32]. Future studies should investigate the cellular impact of individual metabolite components.

In this study, we conducted cellular experiments based on the hypothesis that vaginal bacteria influence HPV infection in epithelial cells, supported by the observation of a significantly higher prevalence of vaginal *Gardnerella* in women with HPV infection. The expected outcome of the experiment was an increase in membrane-bound SDC-1 expression, correlating with increased susceptibility to HPV. Surprisingly, the experimental results contradicted this expectation, failing to provide a plausible explanation for the observed clinical phenomenon. The subsequent extensive literature review led us to discover that SDC-1 can be shed but retains its HPV-binding domain, forming SDC-1-HPV complexes in the extracellular matrix [[11], [12], [13], [14]]. These complexes can swiftly penetrate cells lacking SDC-1 expression, potentially reaching the nucleus. Consequently, the decrease in membrane SDC-1 levels and gene expression may reduce the vulnerability of cells with limited SDC-1 expression to HPV. However, a surplus of shed SDC-1 in the extracellular matrix could enhance cellular susceptibility to HPV upon exposure. This contradicts previous notions that sustained reduction in SDC-1 expression and membrane-bound SDC-1 would diminish cellular sensitivity to HPV, underscoring the complexity of the clinical scenario.

This study has certain limitations. Firstly, the study only utilized one cell line, highlighting the need for validation with primary cervical epithelial cells for broader applicability. Secondly, the intervention using metabolites from three vaginal bacteria strains lacked comparison with live or heat-inactivated bacteria controls. Additionally, no pH adjustment was made for the LCS. Thirdly, this study solely investigated the impact of a single bacterial strain on SDC-1 in cervical epithelial cells, neglecting the complexity of the vaginal microbiota. Fourthly, this study was limited to monolayer cell experiments, disregarding cellular differentiation levels. Fifthly, this study lacks molecular mechanism research, hindering a comprehensive understanding of cervical epithelial tissue structure. Sixthly, the variations in metabolite concentrations may potentially affect the study results. Future studies are warranted.

## 5 Conclusion

This study demonstrates that LCS enhances the proliferation of HcerEpic cells, induces the secretion of pro-inflammatory factors at lower levels, and does not significantly impact the expression and shedding of SDC-1, providing molecular-level evidence for its role as a protective factor against HPV infection in the vagina. On the other hand, metabolites of GV and AV impede the proliferation of HcerEpic cells, promote the secretion of pro-inflammatory factors, inhibit the transcription and expression of SDC-1, and facilitate SDC-1 shedding. Consequently, this study establishes a theoretical foundation for heightened vulnerability to cervical HPV infection in a disturbed vaginal microenvironment. It also suggests that modulating the vaginal microenvironment may have the potential to prevent cervical HPV infection, thus offering a theoretical basis for future clinical translational research.

### Ethical statement

Not applicable.

## Funding

This study was funded by the 10.13039/501100015310Xinjiang Uygur Autonomous Region Natural Science Foundation General Project (2022D01A314), and the Open Project of the State Key Laboratory of Pathogenesis, Prevention and Treatment of High Incidence Diseases in Central Asia (SKL-HIDCA-2020-GJ1)

## Data availability statement

The data that support the findings of this study are available from the corresponding author upon reasonable request.

## CRediT authorship contribution statement

**Yan Xia:** Writing – original draft, Visualization, Software, Methodology, Investigation, Funding acquisition, Formal analysis, Data curation. **Ying Feng:** Writing – original draft, Software, Investigation, Conceptualization. **Lan Jiang:** Writing – review & editing, Methodology, Investigation. **Youqiang Heng:** Methodology, Investigation. **Xiaoqin Li:** Writing – review & editing, Conceptualization. **Cailing Ma:** Writing – review & editing, Supervision, Project administration, Conceptualization.

## Declaration of competing interest

The authors declare that they have no known competing financial interests or personal relationships that could have appeared to influence the work reported in this paper.
